# Response of rapeseed growth to soil salinity content and its improvement effect on coastal saline soil

**DOI:** 10.3389/fpls.2025.1601627

**Published:** 2025-08-04

**Authors:** Haoming Wang, Yiyang Li, Yihang Huang, Yan Wang, Wenting Qu, Yaowei Lin, Long Wang, Guobing Lin, Qingsong Zuo

**Affiliations:** ^1^ Agricultural College, Yangzhou University, Yangzhou, China; ^2^ Jiangsu Key Laboratory of Crop Genetics and Physiology, Yangzhou University, Yangzhou, China; ^3^ Jiangsu Co-Innovation Center for Modern Production Technology of Grain Crops, Yangzhou University, Yangzhou, China; ^4^ Key Laboratory of Saline-Alkali Soil Improvement and Utilization (Coastal Saline-Alkali Lands), Ministry of Agriculture and Rural Affairs, Yangzhou, China

**Keywords:** rapeseed, saline soil remediation, soil aggregate, soil nutrients, soil enzyme

## Abstract

Coastal saline soil is considered an important land source due to their abundant thermal and light conditions, irrigation resources, and relatively low reclamation difficulty. However, it is crucial to establish effective strategies for ameliorating saline soil to render it suitable for crop growth and development. As an economic crop with strong salt tolerance, rapeseed (*Brassica napus* L.) may be a pioneer crop for the development and utilization of saline-alkali lands. To explore the adaptability of rapeseed in coastal saline soils and its potential for soil improvement, this study conducted rapeseed cultivation experiments in soils with different salinity levels over three consecutive years. Prior to sowing in the first season, the initial soil salinity levels were measured at 2.49 g kg^−1^ (low-salinity soil, LS) and 4.27 g kg^−1^ (high-salinity soil, HS). The seed yield and biomass of rapeseed, soil physiochemical properties, and soil enzyme activity were investigated. The results revealed that the seed yield and biomass of rapeseed in high-salinity soil were significantly reduced by 40.30% and 30.58% across three growing seasons, compared to low-salinity soil. As the cultivation year progressed, the seed yield and biomass gradually increased. After three years of rapeseed cultivation, total salt content reduced from 2.50–4.20 g kg^−1^ to 1.59–2.79 g kg^−1^, and EC decreased from 0.95–1.38 ms cm^−1^ to 0.32–0.40 ms cm^−1^. Compared to bare land, rapeseed cultivation exhibited a reduction in soil bulk density, along with an increase in porosity and proportions of macro- and micro-aggregates. In terms of chemical properties, after rapeseed cultivation, the contents of organic matter, dissolved organic C, total N, available N, total phosphorus, available phosphorus increased by 56.99%, 10.49%, 47.13%, 64.43%, 19.30%, and 74.31% in the low-salinity soil; correspondingly, the increases in the high-salinity soil were 22.83%, 3.57%, 8.81%, 22.96%, 11.81%, and 53.82%. In addition, rapeseed cultivation augmented the activity of β-glucosidase, urease, protease, and alkaline phosphatase in both low-salinity and high-salinity soils. Overall, rapeseed proved to be an appropriate crop for the remediation of coastal saline soil, effectively ameliorating soil quality by reducing salinity, fortifying soil structure, accumulating nutrients, and fostering soil enzyme activity.

## 1 Introduction

Ensuring the sustainable development of agriculture is critical for meeting the food demands of a growing global population. However, the availability of arable land for agricultural production has been declining due to the over-expansion of urbanization and industrialization (Anwar et al., 2023). Consequently, developing reserve land resources has become an urgent priority to safeguard agricultural production. Natural coastal saline soils are widely regarded as a critical arable land source, given their abundant thermal and light conditions, irrigation resources, and relatively low reclamation difficulty. In China, there are long coastlines and rich tidal flats. Specifically, the North-Jiangsu Plain stands out with 954 km of coastline and 6.67×10^5^ ha of coastal saline soil, accounting for one-quarter of the total such saline soil in China. This region exemplifies the potential for utilizing coastal saline soils to address land scarcity and support sustainable agricultural development.

Natural coastal saline soils are characterized by high salt content, strong alkalinity, high electric conductivity (EC), nutrition deficiencies, and low microbial activity, rendering them unsuitable for most conventional crops (Cui et al., 2021; Li et al., 2023). Therefore, it is essential to explore effective strategies for ameliorating saline soil to allow it for agricultural cultivation. In recent decades, a range of management practices have been developed and employed to address saline soil issues and improve soil productivity. These practices can be broadly categorized into four main approaches: leaching, chemical amendments, organic amendments, and phytoremediation. Leaching involves the application of abundant fresh water to flush salts from the topsoil into deeper soil layers (Guo and Liu, 2020; Liu et al., 2023). However, this technique has been restricted because of its excessive consumption of fresh water, detrimental effects on soil structure stability, and negative impact on soil fertility. Amelioration of saline soil through chemical amendments is a technology that supplies calcium (Ca^2+^) to replace sodium (Na^+^) from the cation exchange sites (Li et al., 2012; Zhao et al., 2018). Organic amendments can also increase the dissolution of native calcite and promote the leaching of Na^+^ and soil stability (Mahmoud et al., 2019; Liang et al., 2021). Despite their effectiveness, the high cost associated with these approaches limits their utilization and compels farmers to find alternative strategies.

Phytoremediation of saline soil, a cost-effective and environmentally friendly approach, involves cultivating specific plant species to transform low-quality saline soils into high-quality arable land. This method has been approved to alleviate the soil salinity degree, improve soil structure stability, and enhance both soil fertility and microbial activity by the establishment of vegetation in the saline soil. Several halophyte species (grasses or forests) have shown promise as effective phytoremediation tools. For example, Xia et al. (2019) found that a forest-grass pattern could improve soil structure, increase nutrient levels, and boost microbial populations, recommending mixed tree-shrub-grass systems as a preferred strategy. Similarly, Yang et al. (2021) demonstrated that Tamarix chinensis-grass patterns significantly decreased soil salt content and enhanced the availability of nutrients in the coastal saline soil. Jing et al. (2019) reported that cultivating *Atriplex triangularis* and *Suaeda glauca* facilitated soil desalinization by altering enzyme activities and improving the diversity and richness of bacterial communities. Recent studies have also explored the potential of crop cultivation, often combined with complementary technologies, to improve saline soil quality. For instance, Xu et al. (2020) demonstrated that three years of rice cultivation reduced soil salinity and pH, increased soil organic matter content and alkaline phosphatase activity, and promoted microbial diversity. Additionally, Jaiswal et al. (2022) reported that rice-wheat and rice-mustard cropping systems with organic amendments have been shown to improve saline soil quality by enhancing carbon (C) and nitrogen (N) cycles as well as activity of urease and invertase. Furthermore, Zhang et al. (2022) demonstrated that long-term cotton cultivation combined with stubble return and subsoiling has been effective in decreasing soil salinity, improving soil porosity and aggregate stability, and promoting the soil C cycle. These findings highlight the potential of crop cultivation as effective solutions for saline soil remediation.

Rapeseed is the second-largest oil crop followed by soybean globally (Khan et al., 2021). Rapeseed oil is a vital source of edible oil, biodiesel, and various industrial chemicals (Koutsouki et al., 2016). According to the data from the National Bureau of Statistics, the total rapeseed cultivation area in China was about 6.99 million hectares in 2021, yielding 14.7 million tons of oilseed (National Bureau of Statistic, 2021). According to previous studies, salt stress typically inhibits the phenotype and physiological processes of rapeseed, such as reduced leaf area and plant height, hindered root extension, as well as impaired leaf photosynthesis and nitrogen uptake capacity (Shahzad et al., 2022; Wang et al., 2024). However, rapeseed can counteract salt stress by enhancing antioxidant levels, regulating the synthesis of osmolytes, and modulating endogenous hormone levels (Tian et al., 2022). Therefore, rapeseed is recognized for its moderate tolerance to salt stress conditions and its ability to contribute to soil quality restoration. Its metal enrichment capabilities have been extensively documented, highlighting its potential for remediating contaminated soils (Dhiman et al., 2016). Wang et al. (2022) demonstrated that rapeseed cultivation during a single season could accumulate 39.45-102.24 kg ha^–1^ of Na^+^ in various salinity soils, outperforming other crops such as maize, sorghum, wheat, millet, and soybean. This underscores the significant potential of rapeseed in saline soil remediation. Despite existing studies on the use of rapeseed for coastal saline soil remediation, the underlying mechanisms remain poorly understood. Therefore, based on the previous studies, we conducted a three-year field experiment to expand the understanding of how rapeseed cultivation regulates soil properties. The present study aims to elucidate the effects of rapeseed cultivation on coastal saline soil remediation by examining changes in soil salinity, soil structure, nutrient availability, and enzyme activity at a field scale.

## 2 Materials and methods

### 2.1 Experimental site

This experiment was conducted in Dafeng County, Jiangsu Province, China, during the rapeseed growing season (2021–2022, 2022–2023, and 2023–2024). This region is characterized by a typical subtropical monsoon climate (transition from north subtropical zone to warm temperature zone) with an average precipitation of 1014 mm and a mean average temperature of 14.4 °C. Situated on the Eastern of the Yellow Sea, this area is classified as coastal saline soil. The soil salinity levels were influenced by altitude, distance to the sea, water table, and other environmental factors. The experimental areas were not previously reclaimed, where no crops were cultivated, and only sparse natural suaeda plants grew.

### 2.2 Experimental design

We selected two fields with different soil salinity characterized by total salt contents of 2.49 g kg^–1^ (low-salinity soil, LS) and 4.27 g kg^–1^ (high-salinity soil, HS), respectively. The soil initial basal properties were listed in **Table 1**. There are four treatments in this experiment: low-salinity soil without rapeseed cultivation (CK1), high-salinity soil without rapeseed cultivation (CK2), low-salinity soil with rapeseed cultivation (T1), and high-salinity soil with rapeseed cultivation (T2). The rapeseed seeds were sown in October each year, and the density was adjusted to 45×10^4^ plants ha^–1^ at the fourth leaf stage. The basal fertilizer was applied with 166.0 kg ha^–1^ of urea, 326.1 kg ha^–1^ of diammonium hydrogen phosphate compound fertilizer, 144.2 kg ha^–1^ of potassium sulfate fertilizer, and 4.5 kg ha^–1^ of boron fertilizer, respectively. In addition, 293.5 kg ha^–1^ of urea was applied as bolting fertilizer. The maturity canola plants were harvested in May of the following year. This study adopted a rapeseed monoculture system, where all rapeseed straw was incorporated into the soil after harvest, with no other crops cultivated during the experimental period.

**Table 1:** The initial soil properties in the topsoil (0-20cm) in 2021.

Plot	Organic matter (g kg^-1^)	AN (mg kg^–1^)	AP (mg kg^–1^)	AK (mg kg^–1^)	Total salt (g kg^–1^)
LS	10.1	60.3	29.4	310	2.49
HS	9.8	57.2	23.5	392	4.27

AN, available nitrogen; AP, available phosphorus; AK, available potassium. LS, low-salinity soil; HS, high-salinity soil.

### 2.3 Sampling and measurement

#### 2.3.1 Soil sampling and measurement

Before the harvest of rapeseed at the third growing season (May 2024), the sampling work was conducted on the consecutive sunny days to avoid the short-term effects of rainfall on soil properties. The soil samples of CK1 and CK2 were derived from the bare land of low-salinity and high-salinity soil, respectively. The soil samples of T1 and T2 were from the plant root zone of low-salinity and high-salinity soil, respectively. Soil samples of each treatment were randomly collected from five points (0–20 cm soil depth) to mix fully into a composite sample. All the soil samples were transported immediately to laboratory in the sterile plastic bags on the dry ice.

Bulk density and soil porosity were determined using the cutting ring method. Soil porosity was calculated by the equation of (1–bulk density/soil specific gravity)×100%), where the soil specific gravity is 2.65 g cm^–3^ (Aziz et al., 2013). Soil aggregates were separated into three size fractions, namely macro-aggregates (250-2000 μm), micro-aggregates (53-250 μm), and silt+clay (< 53 μm) using the wet sieving method with a Laser Diffraction Particle Size Analyzer (Mastersizer 3000, Malvern Panalytical, England). Soil pH was measured employing the pH meter with a water-to-soil ratio of 2.5:1. The EC was measured using the conductivity meter with a water-to-soil ratio of 5:1. Soil total salt was determined by the gravimetric method. The soil total carbon (TC) and total nitrogen (TN) content were quantified using elemental analyzer (Vario EL, Elementar, Langenselbold, Germany). The total organic carbon and dissolved organic carbon (DOC) were extracted with deionized water and subsequently analyzed by the TOC analyzer. The soil organic matter content was 1.724 times of the TOC content. The available nitrogen (AN) was determined using the alkaline hydrolysis diffusion method. The total phosphorus (TP) and available phosphorus (AP) were extracted by NaOH fusion and NaHCO_3_, respectively, and then measured by the molybdenum blue colorimetry method. The total potassium (TK) and available potassium (AK) were extracted by hydrofluoric acid and nitric acid, respectively, and then measured using the flame photometry.

According to the methods of (Zhou and Zhang, 1980), β-Glucosidase activity was determined using the colorimetric method with β-glucoside as the substrate, with one unit of enzyme activity defined as the amount that produced 1 μmol of p-nitrophenol (p-NP) per gram of soil per 24 hours (μmol p-NP g^−1^ 24h^−1^). Invertase activity was determined using the titration method with sucrose as the substrate, with one unit of enzyme activity defined as the amount that produced 1 mg of reducing sugars per gram of soil per 24 hours (mg reducing sugar g^−1^ 24h^−1^) (Mishra et al., 1979). According to the methods of (Tabatabai and Bremner, 1972), urease activity was determined using the sodium phenolate colorimetric method with urea as the substrate, with one unit of enzyme activity defined as the amount that produced 1 μg of NH_3_-N per gram of soil per 24 hours (μg NH_3_-N g^−1^ 24h^−1^). Following the methods of (Saviozzi et al., 2011), protease activity was determined using the ninhydrin colorimetric method with casein as the substrate, with one unit of enzyme activity defined as the amount that produced 1 μg of NH_3_-N per gram of soil per 24 hours (μg NH_3_-N g^−1^ 24h^−1^). Alkaline phosphatase activity was determined using the colorimetric method with disodium phenyl phosphate as the substrate, with one unit of enzyme activity defined as the amount that released 1 mg of P_2_O_5_ per gram of soil per 24 hours (mg P_2_O_5_ g^−1–^24 h^−1^), using the methods of (Tabatabai, 1982).

#### 2.3.2 Plant sampling and measurement

The plant sample was collected at the maturity stage (May) during each growing season. Ten plants were collected randomly in each plot, manually threshed, and then dried at 80 °C to constant weight to determine the dry weight. The biomass was calculated by multiplying the dry weight per plant by density. Moreover, 2×6 m^2^ area was chosen for the measurement of seed yield.

### 2.4 Statistical analysis

The data was first compiled with Microsoft Excel. For the rapeseed plant section (i.e., yield and biomass), encompassing six groups (three years × two soil salinity levels), multiple comparisons were performed using the least significant difference (LSD) test at p=0.05 level. For the soil section, encompassing four groups (bare land of low-salinity soil (CK1), low-salinity land treated with rapeseed cultivation (T1), bare land of high-salinity soil (CK2), and high-salinity land treated with rapeseed cultivation (T2)), multiple comparisons were likewise conducted using the LSD test at p=0.05 level. The data was shown as means of values from three replicates.

## 3 Results

### 3.1 Seed yield and biomass

**Figure 1:**
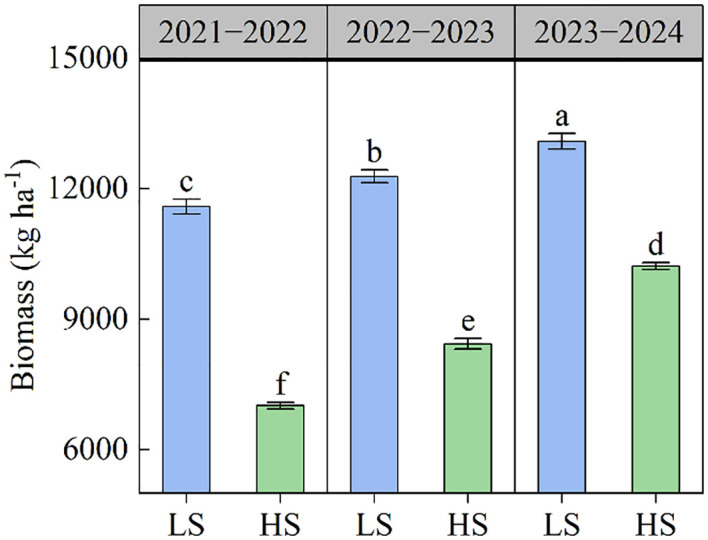
The biomass of rapeseed under different soil salt conditions. Different letters indicate significant differences at *p* = 0.05. LS and HS represent low-salinity soil and high-salinity soil, respectively. Data was presented as means ± standard deviation (n=3).

The results of seed yield and yield components were shown in **Table 2**. The seed yield ranged from 1399.5 to 3460.8 kg ha^−1^ across different treatments. The increase in soil salinity caused a significant reduction in seed yield. Compared to LS soil, the seed yield in HS soil averagely decreased by 40.30% across three growing seasons. The number of pods in population varied from 27.89×10^6^ to 62.68×10^6^ ha^−1^ under different treatments. The number of pods in population in HS soil was 36.80% lower than in LS soil. The number of seeds per pod significantly decreased as soil salinity increased. The average values in LS and HS soils were 15.6 and 14.8 respectively. There was no significant difference in 1000-seed weight between LS soil and HS soil. As the cultivation year progressed, the seed yield, number of pods in population, and number of seeds per pod gradually increased. Similarly, the increase in soil salt content reduced biomass accumulation at maturity stage, whereas the biomass improved as the cultivation year increased (**Figure 1**).

**Table 2:** The seed yield and yield components under different soil salt conditions.

Year	Soil salinity	Seed yield (kg ha^–1^)	Number of pods in population (×10^6^ ha^–1^)	Number of seeds per pod	1000-seed weight (g)
2021–2022	LS	2834.6 c	52.89 c	15.4 c	3.764 a
	HS	1399.5 f	27.89 f	14.5 f	3.753 a
2022–2023	LS	3152.8 b	58.21 b	15.7 b	3.770 a
	HS	1869.3 e	36.31 e	14.8 e	3.763 a
2023–2024	LS	3460.8 a	62.68 a	15.9 a	3.774 a
	HS	2372.1 d	45.64 d	15.1 d	3.767 a

Different letters within the same column indicate significant differences at *p* = 0.05. LS and HS represent low-salinity soil and high-salinity soil, respectively. Data was presented as means (n=3).

### 3.2 Soil physical properties

**Figure 2:**
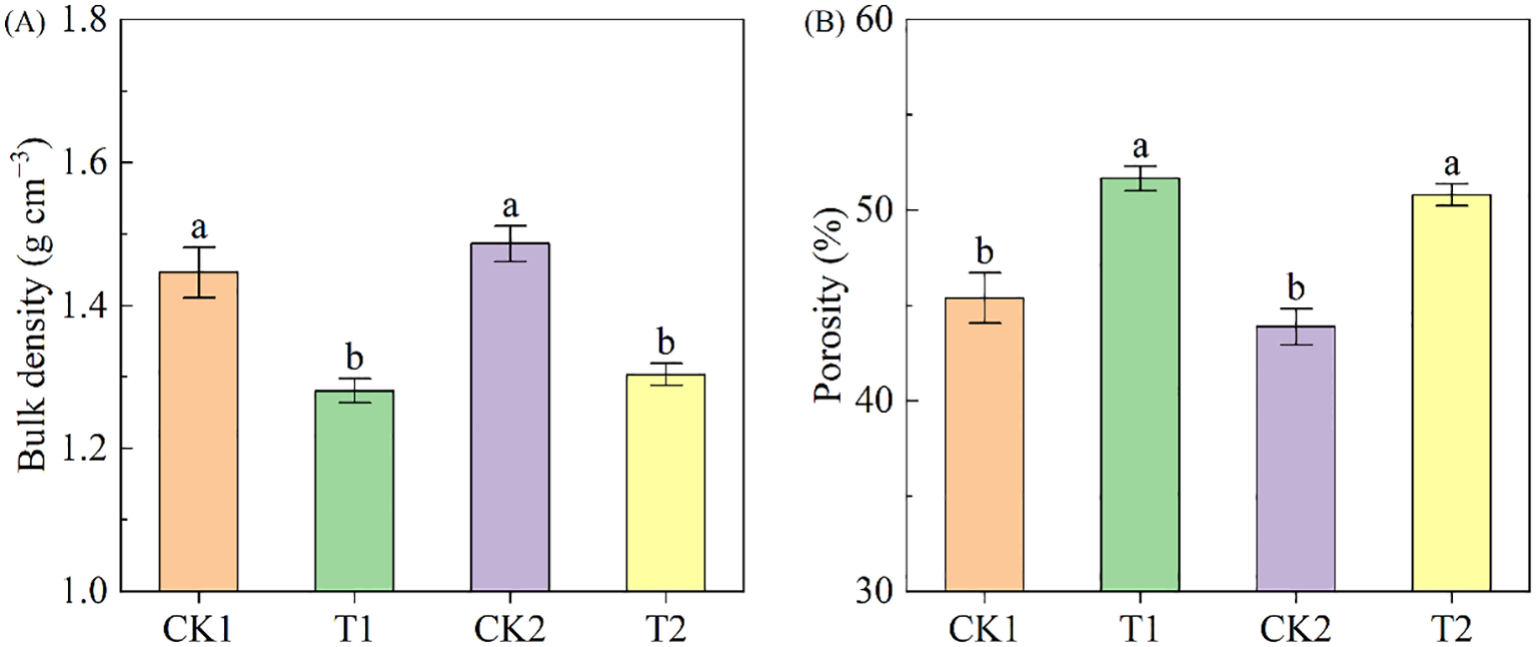
The soil bulk density and porosity under different treatments. **(A)** bulk density; **(B)** porosity. Different letters indicate significant differences at *p* = 0.05. CK1 and CK2 represent bare land of low-salinity soil and high-salinity soil, respectively. T1 and T2 represent low-salinity land and high-salinity land treated with rapeseed cultivation, respectively. Data was presented as means ± standard deviation (n=3).

Compared to bare land, land with rapeseed cultivation exhibited a significant reduction in soil bulk density (**Figure 2A**). In the low-salinity soil group, Bulk density under T1 treatment was 11.47% lower than it under CK1 treatment; correspondingly, in the high-salinity soil group, it under T2 treatment was 12.33% lower than under CK2 treatment. Rapeseed cultivation enhanced soil porosity, exhibiting increases of 13.80% and 15.76% in low-salinity soil and high-salinity soil, respectively (**Figure 2B**).

The results of soil aggregate composition under different treatments were demonstrated in **Table 3**. The proportions of silt and clay were the highest among different aggregates, followed by macro-aggregate, the proportion of micro-aggregate was lowest. Rapeseed cultivation altered soil aggregate composition. Rapeseed cultivation increased the proportions of macro-aggregate and micro-aggregate, while reduced the proportions of silt and clay, in both low salt-salinity and high-salinity soils.

**Table 3:** The soil aggregate composition in different treatments.

Treatment	Macro-aggregate (%)	Micro-aggregate (%)	Silt+clay (%)
CK1	6.09 b	32.56 c	61.35 b
T1	8.62 a	42.19 a	49.19 d
CK2	3.68 d	30.98 d	65.34 a
T2	4.93 c	40.60 b	54.46 c

Different letters within the same column indicate significant differences at *p* = 0.05. CK1 and CK2 represent bare land of low-salinity soil and high-salinity soil, respectively. T1 and T2 represent low-salinity land and high-salinity land treated with rapeseed cultivation, respectively. Data was presented as means (n=3).

### 3.3 Soil chemical properties

#### 3.3.1 Soil pH, EC, and total salt

**Figure 3:**
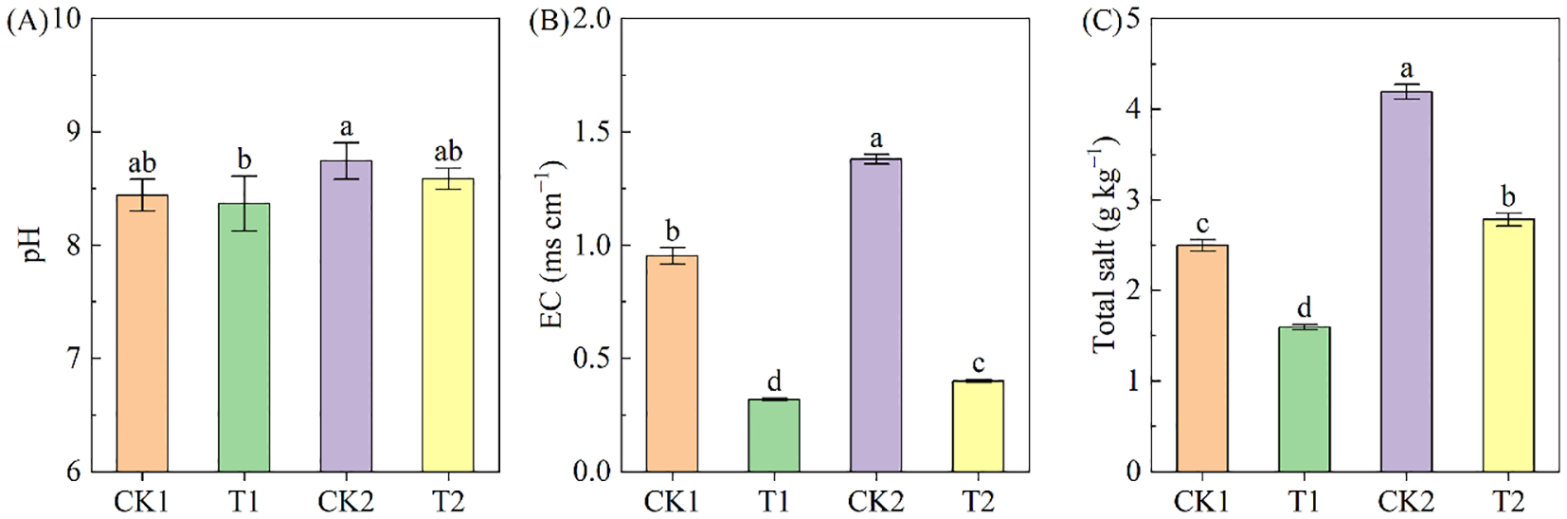
The soil pH, EC and total salt under different treatments. **(A)** pH; **(B)** EC; **(C)** total salt. Different letters indicate significant differences at *p* = 0.05. CK1 and CK2 represent bare land of low-salinity soil and high-salinity soil, respectively. T1 and T2 represent low-salinity land and high-salinity land treated with rapeseed cultivation, respectively. Data was presented as means ± standard deviation (n=3).

Soil pH ranged from 8.37 to 8.75 across treatments, with the order CK2>T2>CK1>T1. Rapeseed cultivation resulted in a small but non-significant decrease in pH in both soil types compared to controls (**Figure 3A**). The EC and total salt varied from 0.32 to 1.38 ms cm^–1^ and 1.59 to 4.20 g kg^–1^ across different treatments, respectively (**Figures 3B, C**). In both bare land and rapeseed cultivation land, the EC and total salt in high-salinity soil were significantly higher than those in low-salinity soil. Rapeseed cultivation significantly reduced soil EC and total salt. In the low-salinity soil group, the EC and total salt under T1 treatment were 66.38% and 36.15% lower than under CK1 treatment; correspondingly, in the high-salinity soil group, the EC and total salt under T2 treatment were 70.91% and 33.59% lower than under CK2 treatment.

#### 3.3.2 C and N nutrient availability

The results of soil C and N nutrients under different treatments were shown in **Table 4**. The TC content varied from 17.35 to 20.26 g kg^–1^ under different treatments. In the low-salinity soil group, T1 treatment increased soil TC content by 16.81% compared to CK1 treatment. However, rapeseed cultivation had no significant effect on TC content in the high-salinity soil group. The order of organic matter and DOC content followed: T1>T2>CK1>CK2. In the low-salinity soil group, T1 treatment increased organic matter and DOC content by 56.99% and 10.49% respectively, compared to CK1 treatment; correspondingly, in the high-salinity soil group, T2 treatment increased organic matter and DOC content by 22.83% and 3.57% respectively. Concurrently, the TN and AN content varied from 0.75 to 1.12 g kg^–1^ and 53.12 to 92.17 mg kg^–1^, respectively. Rapeseed cultivation led to a notable increase in TN and AN. In the low-salinity soil group, T1 treatment resulted in an increase of 47.13% in TN and 64.43% in AN, respectively, compared to CK1; in the high-salinity soil group, the corresponding increases were 8.81% and 22.96%, respectively.

**Table 4:** The soil C and N nutrients in different treatments.

Treat	TC (g kg^–1^)	Organic matter (g kg^–1^)	DOC (mg kg^–1^)	TN (g kg^–1^)	AN (mg kg^–1^)
CK1	17.35 c	9.92 c	191.26 bc	0.76 c	56.05 c
T1	20.26 a	15.57 a	211.33 a	1.12 a	92.17 a
CK2	18.42 b	8.56 d	188.71 c	0.75 c	53.12 d
T2	18.81 b	10.52 b	195.44 b	0.82 b	65.31 b

Different letters within the same column indicate significant differences at *p* = 0.05. CK1 and CK2 represent bare land of low-salinity soil and high-salinity soil, respectively. T1 and T2 represent low-salinity land and high-salinity land treated with rapeseed cultivation, respectively. Data was presented as means (n=3).

#### 3.3.3 P and K nutrient availability

**Figure 4:**
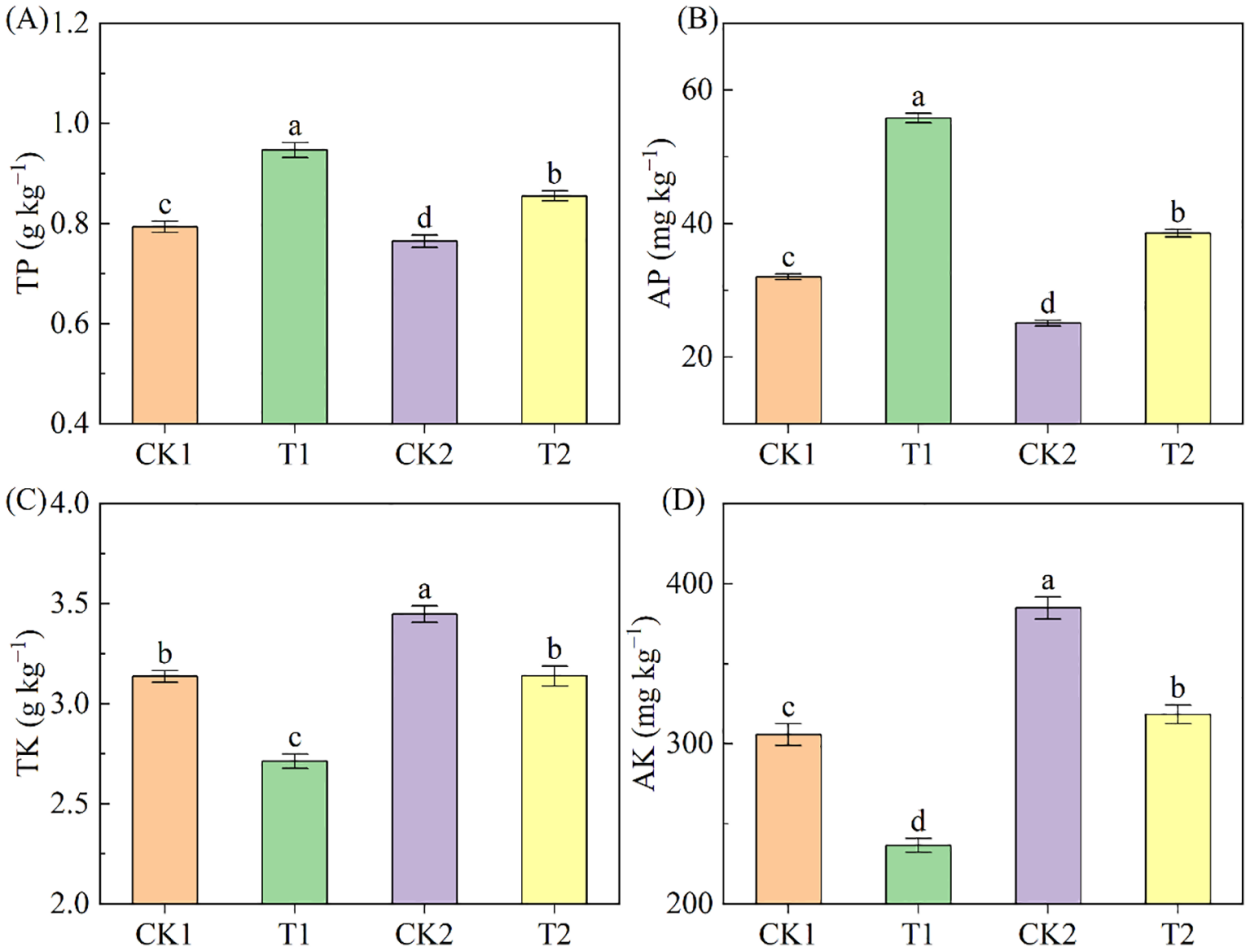
The soil P and K nutrients under different treatments. **(A)** TP; **(B)** AP; **(C)** TK; **(D)** AK. Different letters indicate significant differences at *p* = 0.05. CK1 and CK2 represent bare land of low-salinity soil and high-salinity soil, respectively. T1 and T2 represent low-salinity land and high-salinity land treated with rapeseed cultivation, respectively. Data was presented as means ± standard deviation (n=3).

The contents of TP and AP in the low-salinity soil were higher than in the high-salinity soil and significantly elevated after rapeseed cultivation, followed the order: T1>T2>CK1>CK2 (**Figures 4A, B**). In the low-salinity soil group, T1 treatment augmented TP and AP by 19.30% and 74.31%, respectively, compared to CK1; in the high-salinity soil group, T2 treatment increased TP and AP by 11.81% and 53.82% respectively, compared to CK2. Additionally, the contents of TK and AK in the low-salinity soil were lower than those in the high-salinity soil, and rapeseed cultivation resulted in a drop in soil TK and AK contents (**Figures 4C, D**). Compared to CK1, T1 treatment induced a decrease of 13.51% and 22.64% in TK and AK; compared to CK2, T2 treatment reduced 8.96% and 17.30% in TK and AK respectively.

### 3.4 Enzyme

**Figure 5:**
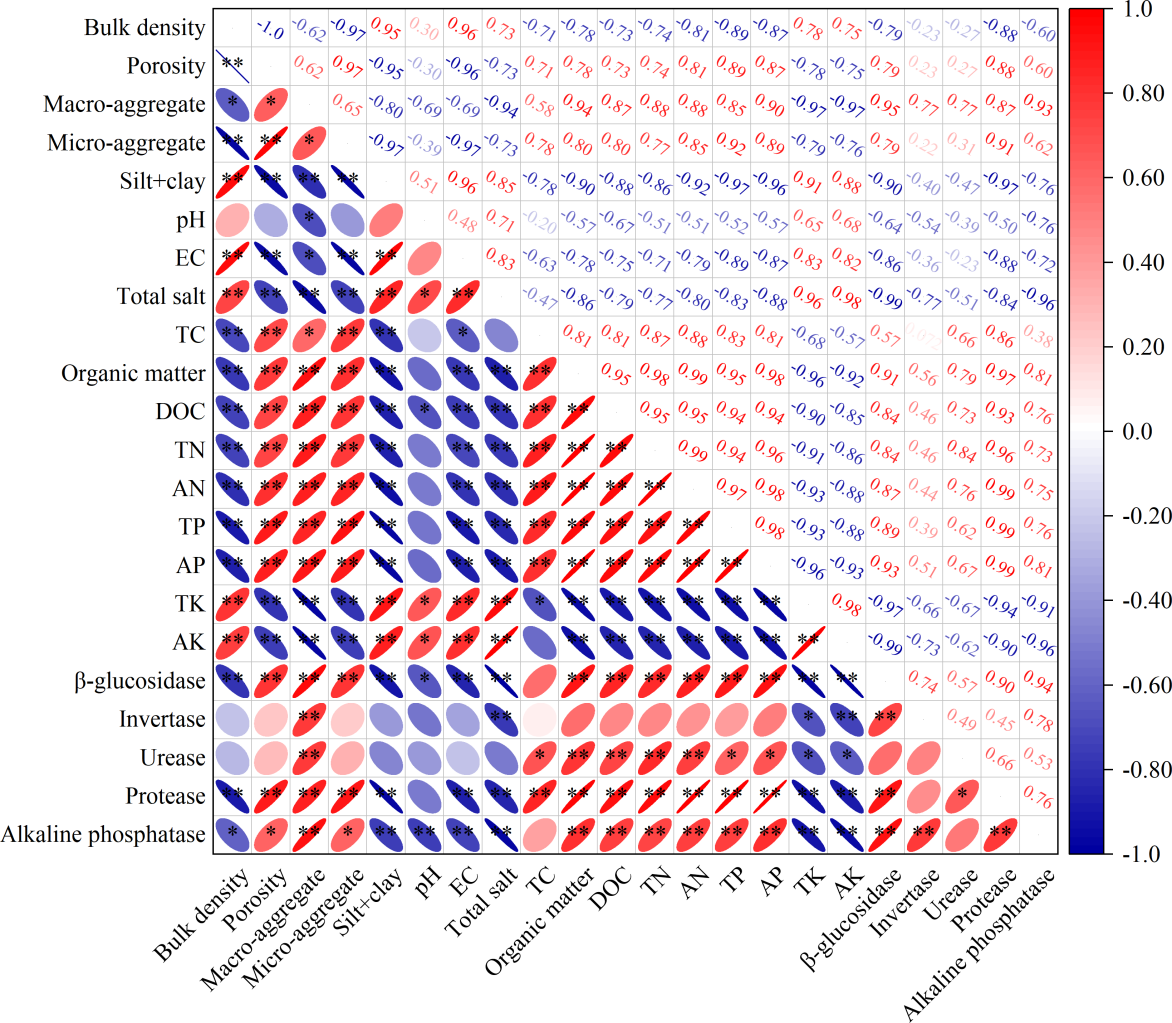
Pearson’s correlation analysis of soil physicochemical properties. Probability levels are indicated by * and ** for 0.05 and 0.01, respectively.

The results of activity of β-glucosidase, invertase, urease, protease, and alkaline phosphatase were documented in **Table 5**. β-glucosidase activity under different treatments followed the order: T1>CK1>T2>CK2. Rapeseed cultivation significantly improved β-glucosidase activity. In the low-salinity soil group, β-glucosidase activity under T1 treatment was 31.24% higher than under CK1 treatment; in the high-salinity soil group, β-glucosidase activity under T2 treatment was 55.25% higher than under CK2. Invertase activity in low-salinity soil group was significantly higher than in high-salinity soil group. However, no significant effect of rapeseed cultivation on invertase activity was observed. The effect of rapeseed cultivation on urease activity varied between low-salinity soil and high-salinity soil. In the low-salinity soil group, T1 treatment increased urease activity by 47.41% compared to CK1 treatment; however, in the high-salinity soil group, urease activity under T2 treatment was significantly lower than under CK2 treatment. Protease activity under different treatments followed the order: T1>T2>CK1>CK2. Rapeseed cultivation increased protease activity by 121.45% in the low-salinity soil group and 82.18% in the high-salinity soil group respectively. The soil alkaline phosphatase activity under different treatments varied from 74.17 to 83.05 mg P_2_O_5_ g^–1^ 24h^–1^, following the order: T1>CK1>T2>CK2 (**Figure 5**). Rapeseed cultivation significantly elevated soil alkaline phosphatase activity in both low-salinity soil and high-salinity soil. In the low-salinity soil group, T1 treatment increased alkaline phosphatase activity by 3.01% compared to CK1 treatment; in the high-salinity soil group, the soil alkaline phosphatase activity under T2 treatment was 5.60% higher than under CK2 treatment.

**Table 5:** The activity of enzyme in saline soil under different treatments.

Treatment	β-glucosidase (μmol p-NP g^–1^ 24h^–1^)	Invertase (mg reducing sugar g^–1^ 24h^–1^)	Urease (μg NH_3_-N g^–1^ 24h^–1^)	Protease (μg NH_3_-N g^–1^ 24h^–1^)	alkaline phosphatase (mg P_2_O_5_ g^–1^ 24h^–1^)
CK1	4.11 b	6.05 a	407.78 c	7.98 c	80.62 b
T1	5.40 a	6.02 a	601.11 a	17.67 a	83.05 a
CK2	2.60 c	5.55 b	431.51 b	6.27 d	74.17 d
T2	4.04 b	5.64 b	322.21 d	11.42 b	78.32 c

Different letters within the same column indicate significant differences at *p* = 0.05. CK1 and CK2 represent bare land of low-salinity soil and high-salinity soil, respectively. T1 and T2 represent low-salinity land and high-salinity land treated with rapeseed cultivation, respectively. Data was presented as means (n=3).

### 3.5 Relationship analysis

The relationship analysis (**Figure 5**) showed that soil EC and total salt were negatively related to the contents of TC, organic matter, DOC, TN, AN, TP, and AP, and the activity of β-glucosidase, protease, and alkaline phosphatase. Moreover, the soil β-glucosidase activity was positively related to content of soil organic matter and DOC. The activity of soil protease and urease was positively related to content of soil TN and AN. The activity of alkaline phosphatase was positively related to content of soil TP and AP.

## 4 Discussion

### 4.1 Effects of soil salinity on rapeseed production and soil utilization

Rapeseed is the major source of vegetable oil in China, and seed yield serves as a critical indicator of crop adaptability to salt stress. While low soil salinity has minimal impact on rapeseed seed yield, a significant decline occurs when soil salinity exceeds a certain threshold. For example, Steppuhn et al. (2010) reported that the seed yield of rapeseed was mostly unaffected within an EC range of 1.36–6.05 dS m^−1^, producing a seed yield of 240–254 g m^−2^; however, the seed yield was markedly decreased to 23.78 g m^−2^ when EC increased to 23.78 dS m^−1^. In this study, the average seed yield in low-salinity soil across three growing seasons was 3149.4 kg ha^−1^, whereas the yield in high-salinity soil significantly decreased to 1880.3 kg ha^−1^. Seed yield components, including the number of pods in population, seeds per pod, and 1000-seed weight, exhibit varying responses to salt stress. Our findings indicate that salt stress primarily reduces the number of pods in population, followed by seeds per pod, while having no significant effect on 1000-seed weight. Bybordi (2010) similarly observed that salt stress dramatically reduced the number of pods in population, whereas seeds per pod and 1000-seed weight remained relatively stable, suggesting that yield loss is predominantly due to a decrease in pod number. Overall, the number of pods in population is highly sensitive to salt stress. Therefore, breeding salt-tolerant rapeseed varieties should prioritize selecting strains with a higher and more stable pod number.

From an agricultural production perspective, when crop yields reach 70%-90% of the regional average yield, they can be considered stable enough to meet production requirements (Grassini et al., 2009). In this study, the average seed yield of rapeseed during the first growing season reached 2834.6 kg ha^−1^ in the low-salinity soil, accounting for 92.16% of the local conventional cropland average [the regional average seed yield in Dafeng was 3,075 kg ha^−1^ (Wang, 2014)]. This indicates that coastal saline soils with an initial salt content ≤ 2.49 g kg^−1^ can be effectively utilized for rapeseed cultivation. Contrastingly, the seed yield in high-salinity soil was significantly lower than that in conventional farmland. Given the observed improvement in saline soil properties after three years of rapeseed cultivation, coastal saline soils with salt content exceeding 4.27 g kg^−1^ should prioritize remediation, with rapeseed cultivation serving as an effective strategy for soil reclamation.

### 4.2 Response of saline soil physical structure and salt content to rapeseed cultivation

Saline soils are typically characterized by the degradation of physical structure, deficiencies in chemical nutrients, and suppressed microbial activity. Previous studies have demonstrated that plant cover plays a significant role in the restoration of soil ecosystems (Jesus et al., 2015; Zheng et al., 2023). Bulk density and porosity are critical indicators of soil physical properties, with lower bulk density indicating looser soil texture and well-developed soil pores. In this study, after rapeseed cultivation, significant improvements were observed in soil physical properties, including an 11.47%–12.33% reduction in bulk density, a 13.80%–15.76% increase in porosity, and a notable enhancement in soil aggregate proportion. These improvements are primarily attributed to the synergistic effects of tillage practices and crop cultivation. Initial plowing operations effectively break up the dense compacted layer of saline-alkali soil, improving soil aeration and water permeability, thereby establishing a foundational improvement for subsequent phytoremediation (Liang et al., 2011). Moreover, organic substances such as sugars, proteins, and humus secreted by rapeseed roots promote the adhesion of soil particles, thereby facilitating the formation and stabilization of aggregates (Tan and Kang, 2009).

In the coastal saline soil, the domain saline ions are Na^+^, Ca^2+^, and Cl^−^. The high content of Na^+^ in soil tends to combine with HCO_3_^−^ to form NaHCO_3_, leading to increased soil alkalinity. Typically, the pH of saline soil exceeds 8.5, and can surpass 10 in severe cases. In this experiment, the pH of coastal saline soils ranged from 8.44 to 8.75, which is lower than that typically observed in inland saline soils. The elevated concentration of saline ions also resulted in high EC value. After three years of rapeseed cultivation, both EC and total salt content in saline soil were significantly decreased, consistent with the ameliorative effects reported for other crops (Abiala et al., 2018; Wang et al., 2019; Zheng et al., 2023). Notably, no significant difference in soil pH was recorded between bare land and rapeseed-cultivated land, likely due to the relatively short cultivation period and the initially low pH values. In a long-term comparative experiment, a significant reduction in soil pH was observed after more than 9 years of crop cultivation (Singh et al., 2016). Therefore, EC and total salinity measurements are recommended as the reliable indicators for assessing the improvement of coastal saline soils over shorter periods.

### 4.3 Response of saline soil nutrients to rapeseed cultivation

It is acknowledged that plants play an important role in soil C and N stock (Hinsinger et al., 2006). Soil C and N compositions contain inorganic C, organic matter, and available N et al., among which organic matter and available N are key factors determining soil fertility and quality. This study demonstrated that three years of rapeseed cultivation markedly enhanced soil organic matter and available N content, likely attributed to the considerable residues from rapeseed, which include organic C and N compounds such as amino acids, sugars, and proteins, that were returned to and decomposed in the soil. Soil dissolved organic C is a kind of soil active organic C, representing the most dynamic composition of soil organic C, characterized by rapid breakdown and a swift turnover rate. Leifeld and Kögel-Knabner (2005) pointed out that the total soil C pool is relatively stable in the short term, however, its active components are highly responsive to environmental change and agricultural practice. Our study found that after rapeseed cultivation, the total C content remained largely unchanged in the high-salinity soil; however, the dissolved organic C content exhibited a substantial rise. Notably, dissolved organic C is the most readily lost organic C component in soil due to its significant migratory capacity (McTiernan et al., 2001). This study revealed that the increase in dissolved organic C content after rapeseed cultivation was significantly less than the increase in organic matter content, reflecting the inherent susceptibility of dissolved organic C to loss through processes such as leaching and microbial decomposition. Consequently, minimizing the loss of dissolved organic C is crucial for sustaining the effect of saline soil amelioration.

Our preliminary investigation found that coastal saline soil exhibited N and P shortage and K enrichment. Despite the numerical sufficiency of total P in saline soil, the available P content is markedly low in saline soil due to the absorption of P on the surface of Na and Ca (Sharif et al., 2000; Vance et al., 2003). In the present study, rapeseed cultivation significantly elevated the contents of total P and available P, and the correlation analysis further revealed the negative relationship between total salt content and total P and available P contents. During the rapeseed growth period, root exudates and microbial activity may reduce the fixation of Na and Ca with P and promote P release, consequently increasing the P availability in soil. K within a specific range is a vital nutrient for plant growth and development; however, in coastal saline soils, K concentration surpasses the normal level and manifests as salt ions. Bao et al. (2024) reported that the soil K level is associated with soil physical quality, as K can absorb and bind to the surfaces of silt and clay particles, resulting in complex formations. Rapeseed cultivation decreased the proportion of silt and clay particles, hence enhancing downward movement of K ion and reducing the K content in the topsoil.

In the experiment, compared to the high-salinity soil, the low-salinity soil showed a great improvement in the nutrients after rapeseed cultivation. This may be because low-salinity soil has a lower initial salinization level, making it easier to improve. Additionally, rapeseed grown in such soil exhibits greater biomass, which enables it to absorb and retain more nutrients, thereby resulting in a more effective enhancement of soil fertility. Consequently, we suppose that the beneficial impact of rapeseed cultivation on coastal saline soils will diminish as soil salinization intensifies. For the heavily saline soil, it is advisable to integrate additional measures, such as the use of nutrients, irrigation, and salt pressing, or to prolong the cultivation time, which may yield enhanced improvement effect.

### 4.4 The relationship between soil physicochemical properties and enzyme activity

Soil enzyme activities are intimately linked to soil physical structure and chemical composition. Tillage practices and plant growth can effectively improve soil physicochemical properties, thereby modulating enzymatic activities. It was reported that soil environmental factors affect the soil activity of microorganisms and plant root growth, thereby affecting the soil enzyme activity (Cevheri et al., 2022; Erdel, 2022; Che et al., 2023). Soil aggregates serve as the primary dwelling place for microorganisms, where most biochemical reactions occur within these aggregates. In our study, the activities of β-glucosidase, invertase, urease, protease, and alkaline phosphatase were positively related to the ratio of macro-aggregate. Wang et al. (2022) agreed with our results, who reported that the activities of soil invertase and urease increased with the increase in soil particle size, and the highest value was recorded in the 250-2000 μm of aggregates. The pH, EC and total salt content were negatively and significantly related to the activity of β-glucosidase and alkaline phosphatase in the present study, indicating high sensitivity of these enzymes to soil salinity. Rietz and Haynes (2003) reported that irrigation-induced soil salinization significantly inhibits β-glucosidase activity. Pan et al. (2013) further demonstrated a strong negative correlation between β-glucosidase activity and soil EC, proposing this enzyme as a biomarker for saline soil degradation. It was reported that pH directly influences soil phosphatase activity (Dick et al., 2000). Long-term rapeseed cultivation could remove soil salt and alleviate the adverse effects on microorganisms, thus improving these soil enzyme activities.

The soil C and N status affect microorganisms, thereby changing soil enzyme activity by regulating the diversity and richness of soil microorganisms (Liu et al., 2017). This study found that β-glucosidase activity was positively related to the content of organic matter and dissolved organic C. Sinsabaugh et al. (2008) conducted a meta-analysis, also finding that the β-glucosidase activity increased with an increase in organic matter. However, rapeseed cultivation showed no significant effect on soil invertase activity in this experiment. This may be since the degree of soil maturation may still be insufficient. Moreover, this experiment demonstrated a considerable increase in soil urease and protease activities after rapeseed cultivation, with correlation analysis revealing a strong positive association between these enzyme activities and total N and available N content. This aligned with the findings of (Xie et al., 2017), who discovered that as rapeseed cultivation years progressed, urease activity in saline soil markedly increased. The alkaline phosphatase activity is dependent upon the P availability in the soil (Burke et al., 2011). Rapeseed cultivation enhanced the activation and release of P in the soil, resulting in elevated total and available P levels, which subsequently boosted alkaline phosphatase activity.

In conclusion, β-glucosidase, protease, and alkaline phosphatase exhibited the strongest associations with soil physicochemical properties, establishing these enzymes as reliable biomarkers for monitoring saline-alkali soil remediation.

## 5 Conclusion

The coastal saline soil with a salt content below 2.5 g kg^−1^ produced seed yields comparable to those of local conventional farmland, thereby could be directly utilized for large-scale rapeseed cultivation. In contrast, high-salinity soil with a salt content of 4.2 g kg^−1^ exhibited significantly lower yield levels and required remediation practices. Three-years of rapeseed cultivation significantly reduced soil salinity from 2.50–4.20 g kg^−1^ to 1.59–2.79 g kg^−1^ and decreased EC value from 0.95–1.38 ms cm^−1^ to 0.32–0.40 ms cm^−1^. In terms of soil physical structure, rapeseed cultivation resulted in a significant reduction in soil bulk density, an increase in soil porosity, and an enhancement in soil aggregate proportion. Moreover, rapeseed cultivation promoted soil C, N, and P nutrients, including organic matter, dissolved organic C, total N, available N, total phosphorus, available phosphorus. In addition, rapeseed cultivation augmented soil enzyme activity, such as β-glucosidase, urease, urease, protease, and alkaline phosphatase. Therefore, rapeseed cultivation could be an effective approach for the remediation of coastal saline soil.

## Data Availability

Data inquiries can be directed to the corresponding authors. Requests to access the datasets should be directed to qszuo@yzu.edu.cn.
